# Validation of the 8th edition of the UICC/AJCC staging system for nasopharyngeal carcinoma treated with intensity-modulated radiotherapy

**DOI:** 10.18632/oncotarget.19829

**Published:** 2017-08-02

**Authors:** Min Kang, Pingting Zhou, Guisheng Li, Haolin Yan, Guosheng Feng, Meilian Liu, Jinxian Zhu, Rensheng Wang

**Affiliations:** ^1^ Department of Radiation Oncology, The First Affiliated Hospital of Guangxi Medical University, Nanning 530021, Guangxi, P.R. China; ^2^ Department of Radiation Oncology, Liuzhou Worker Hospital, Liuzhou 545000, Guangxi, P.R. China; ^3^ Department of Radiation Oncology, First People’s Hospital of Yulin City, Yulin 537000, Guangxi, P.R. China; ^4^ Department of Radiation Oncology, People’s Hospital of Guangxi Zhuang Autonomous Region, Nanning 530021, Guangxi, P.R. China; ^5^ Department of Radiation Oncology, Affiliated Hospital of Guilin Medical University, Guilin 541000, Guangxi, P.R. China; ^6^ Department of Radiation Oncology, Wuzhou Red Cross Hospital, Wuzhou 543000, Guangxi, P.R. China

**Keywords:** nasopharyngeal carcinoma, intensity-modulated radiotherapy, UICC/AJCC staging system, prognosis, differences

## Abstract

An accurate TNM staging system is crucial for treatment guidance and prognosis prediction in nasopharyngeal carcinoma (NPC) patients. In this retrospective study, we evaluated the 8th edition of the Union for International Cancer Control/American Joint Committee on Cancer (UICC/AJCC) staging system for NPC treated with intensity-modulated radiotherapy (IMRT). A total of 608 patients with biopsy-proven, non-metastatic NPC, treated with IMRT between January 2008 and March 2010, were enrolled. The 5-year overall survival (OS), disease-free survival (DFS), local relapse-free survival (LRFS), and distant metastasis-free survival (DMFS) rates were 81.5%, 80.1%, 86.0%, and 81.1%, respectively. The LRFS rates of patients with stages T1 vs. T2, T2 vs. T3, and T1 vs. T3 did not differ between the 7th and 8th editions. By contrast, the DMFS rates of patients with N0 vs. N1, N1 vs. N2, and N2 vs. N3 differed between the 8th and the 7th editions, though no difference was observed between N3a and N3b, according to the 7th edition. The difference in OS between stages II and III, and between stages III and IVa, was larger according to the 8th edition than the 7th edition. There was no difference in the OS between stages I and II. These data indicate that in the IMRT era, the 8th edition staging system can predict the prognosis of NPC patients more accurately than the 7th edition.

## INTRODUCTION

The recently introduced intensity-modulated radiotherapy (IMRT) has greatly improved diagnosis and treatment of nasopharyngeal carcinoma (NPC) patients, resulting in the 5-year overall survival (OS) rate of 80% [1–6]. However, the 7th edition of the UICC/AJCC staging system published in 2009 [7], is based on the conventional 2D-RT technique, and does not reflect the recent diagnostic and therapeutic advances.

For NPCs, an accurate TNM staging system is crucial for treatment guidance and prognosis prediction. Therefore, it is important to establish an optimal TNM staging system to be able to make accurate prognostic predictions and treatment guidance. At present, the eighth edition of the UICC/AJCC staging system [8], which is a revision based on the seventh edition, and the Chinese 2008 staging system [9], are universally accepted. There are three main adjustments for tumor classifications: (1) T0 is added for Epstein-Barr virus (EBV) positive unknown primary with cervical lymph node involvement; (2) Confirmed the prognostic value of the prevertebral muscle in NPC patients and designated as T2; (3) The previous T4 criteria “masticator space” and “infratemporal fossa” are now replaced by specific description of soft tissue involvement, and tumors invading medial pterygoid muscle (MP) and/or lateral pterygoid muscle (LP) are now down-staged as T2. Other adjustments include merging of N3a and N3b into N3, and inclusion of the supraclavicular fossa criterion, which was changed to include the lower neck in the N category. In addition, the previous sub-stages IVA (T4N0-2M0) and IVb (any T N3, M0) are now merged to form IVa, and the previous IVc (any T any N M1) is now upstaged to IVb. However, these adjustments need to be validated by results from multiple centers and/or large-sample studies [10].

Here, we performed a retrospective study that enrolled 608 NPC patients who were treated with IMRT and staged by magnetic resonance imaging (MRI) at the first affiliated hospital of Guangxi Medical University. We aimed to verify the accuracy of the 8th edition of the UICC/AJCC staging system for the prognosis prediction of NPC.

## RESULTS

### Survival outcomes

The median follow-up period was 53 months (range, 4-76 months). The 5-year OS, DFS, LRFS, and DMFS rates for the whole group were 81.5%, 80.1%, 86.0%, and 81.1%, respectively. At the last follow-up, 99 (16.3%) patients had died, and 62 (10.2%) patients developed disease recurrence; the median duration from the end of the primary treatment to the diagnosis of recurrence was 28.0 months (range, 3.0-60.0). In addition, 86 (14.1%) patients developed distant metastases; the median duration from the end of the primary treatment to the development of distant metastases was 23 months (range, 3.0-60). Both disease recurrence and distant metastases developed in 32 patients (5.3%).

### Distribution balance

As listed in Table 1, in the 7^th^ edition of the UICC staging system, the distribution of the T stages was T1 (2.6%), T2 (11.3%), T3 (33.7%), and T4 (52.3%), while in the 8^th^ edition, the distribution of T1-4 was 2.6%, 19.1%, 43.9% and 34.4%, respectively. The case distributions of the N stages in the N0-2 staging systems were equivalent; 16 (2.6%) patients staged as N3a and 5 (0.8%) patients staged as N3b according to the 7^th^ edition system were classified as N3 according to the 8^th^ edition. As for the comparative distribution of the clinical stages, the percentages of patients in stage I, II, III and IVa according to the 8^th^ edition system versus the 7^th^ edition were 1.3%, 7.5%, 38.2% and 49.5% vs. 1.3%, 15.0%, 46.7% and 35.0%, respectively. Overall, the distribution of the 8^th^ staging system was more balanced than that of the 7^th^ edition.

**Table 1:** Comparison of distribution balance between the 8th and 7th edition UICC/AJCC NPC staging system (n = 608)

		8th Edition UICC/AJCC/system				
		T1	T2	T3	T4	total
**7th Edition UICC/AJCC/system**	T1	16(2.6%)				16(2.6%)
	T2		69(11.3%)			69(11.3%)
	T3			205(33.7%)		205(33.7%)
	T4		47(7.7%)	62(10.2%)	209(34.4%)	318(52.3%)
	total	16(2.6%)	116(19.1%)	267(43.9%)	209(34.4%)	608(100%)
		N0	N1	N2	N3	total
	N0	106(17.4%)				106(17.4%)
	N1		284(46.7%)			284(46.7%)
	N2			197(32.4%)		197(32.4%)
	N3a				16(2.6%)	16(2.6%)
	N3b				5(0.8%)	5(0.8%)
	total	106(17.4%)	284(46.7%)	197(32.4%)	21(3.4%)	608(100%)
		I	II	III	IVA	Total
	I	8(1.3%)				8(1.3%)
	II		46(7.5%)			46(7.5%)
	III			232(38.2%)		232(38.2%)
	IVA		45(7.4%)	64(10.5%)	192(31.6%)	301(49.5%)
	IVB				21(3.4%)	21(3.4%)
	Total	8(1.3%)	91(15.0%)	296(46.7%)	213(35.0%)	608(100%)

### Survival predictive value

#### Local relapse-free survival for T classification

**Figure 1:**
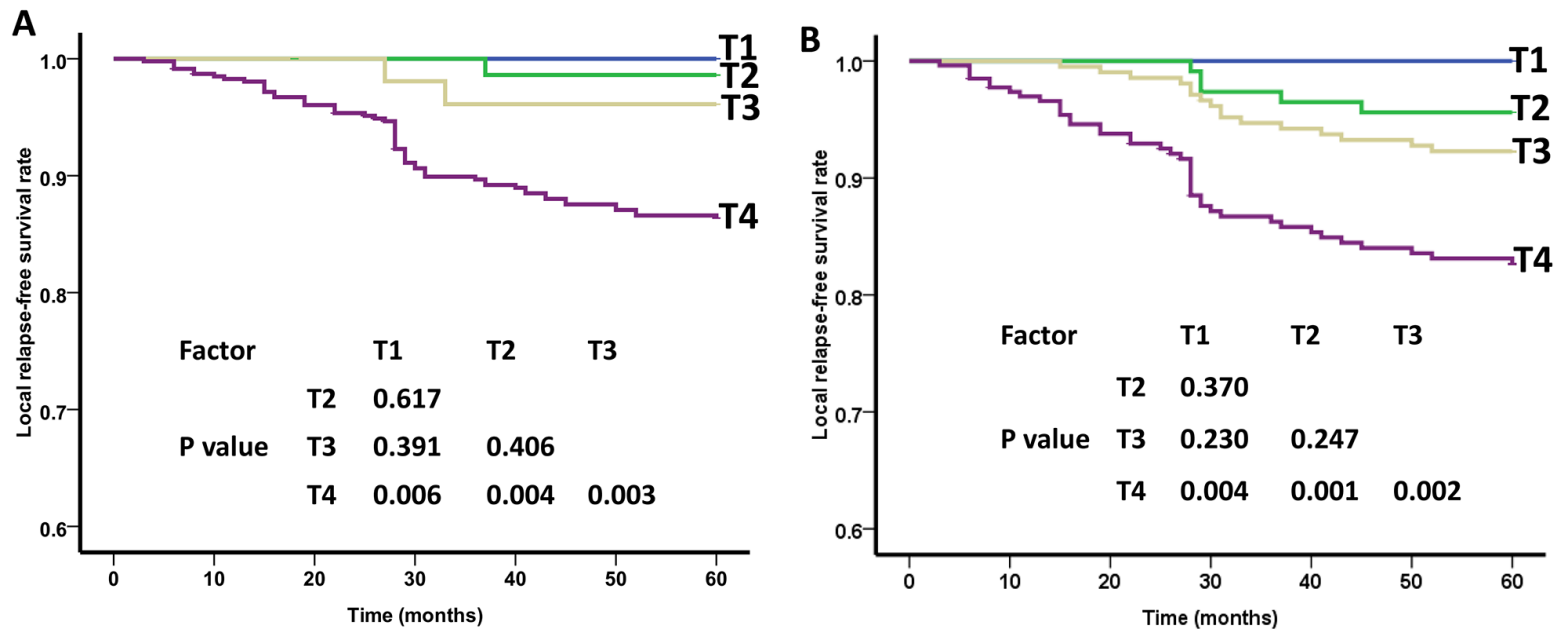
Local relapse-free survival (LRFS) curves of nasopharyngeal carcinoma patients for different T categories **(A)** As defined by the 7th edition UICC/AJCC staging system, the differences in LRFS between T1 -T3 stages were not significant (P>0.05). **(B)** As defined by the 8th edition UICC/AJCC staging system, the differences in LRFS between T1 -T3 stages were not significant (P>0.05).

The 5-year LRFS rates between the 7th and the 8th editions in patients with T1, T2, T3 and T4 were 100% vs. 100%, 98.6% vs. 95.7%, 96.1% vs. 92.3%, and 84.4% vs. 82.7%, respectively. The differences in LRFS between the 7^th^ and the 8^th^ editions in T1 and T2 stages (P = 0.617 vs. P = 0.370), T2 and T3 stages (P = 0.406 vs. P = 0.247), and T1 and T3 stages (P = 0.391 vs. P = 0.230) were not significant. Thus, it seems reasonable to downstage T2-3 to T1 in a future revised edition (Figure 1).

### Distant metastasis-free survival for N classification

**Figure 2:**
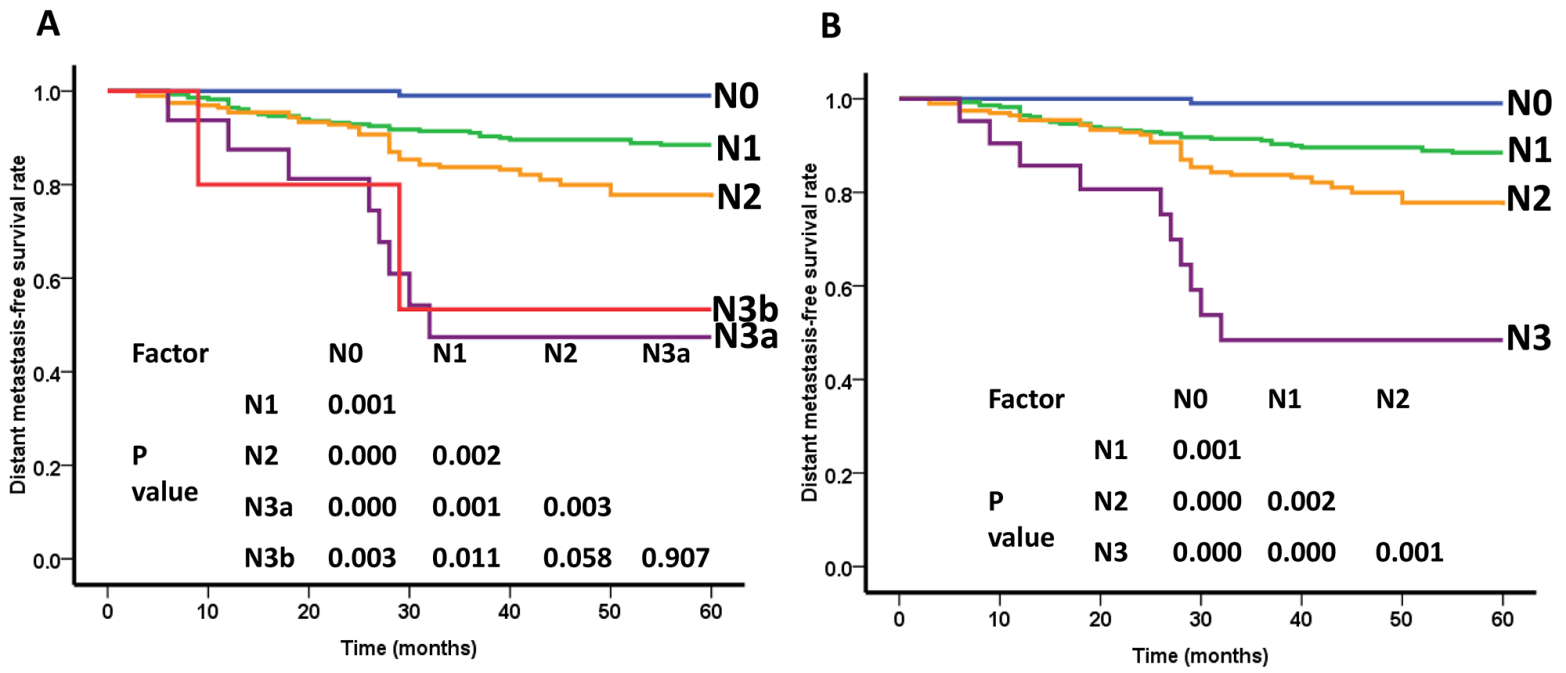
Distant metastasis-free survival(DMFS) curves of nasopharyngeal carcinoma patients for different N categories **(A)** As defined by the 7th edition UICC/AJCC staging system, the DMFS rate between N0 -N3a stages were differed significantly (P<0.05), while, the difference in DMFS between N3a and N3b were not significant (P=0.907). **(B)** As defined by the 8th edition UICC/AJCC staging system, the DMFS rate between N0 -N3 stages were differed significantly (P<0.05).

According to the 8^th^ edition UICC/AJCC staging system, the 5-year DMFS rates for patients with N0, N1, N2, and N3 disease were 99.0%, 88.5%, 77.3%, and 48.4%, respectively. When the 8^th^ edition of the UICC/AJCC staging system was used, the DMFS between N0 and N1, N1 and N2, and N2 and N3 differed significantly (P=0.001, P=0.002, P=0.001). In contrast, no significant difference in survival was observed between N3a and N3b (P=0.907) in the 7^th^ edition, which indicates that the combination of N3a and N3b into N3 is appropriate (Figure 2).

### Overall survival rate for stage grouping

**Figure 3:**
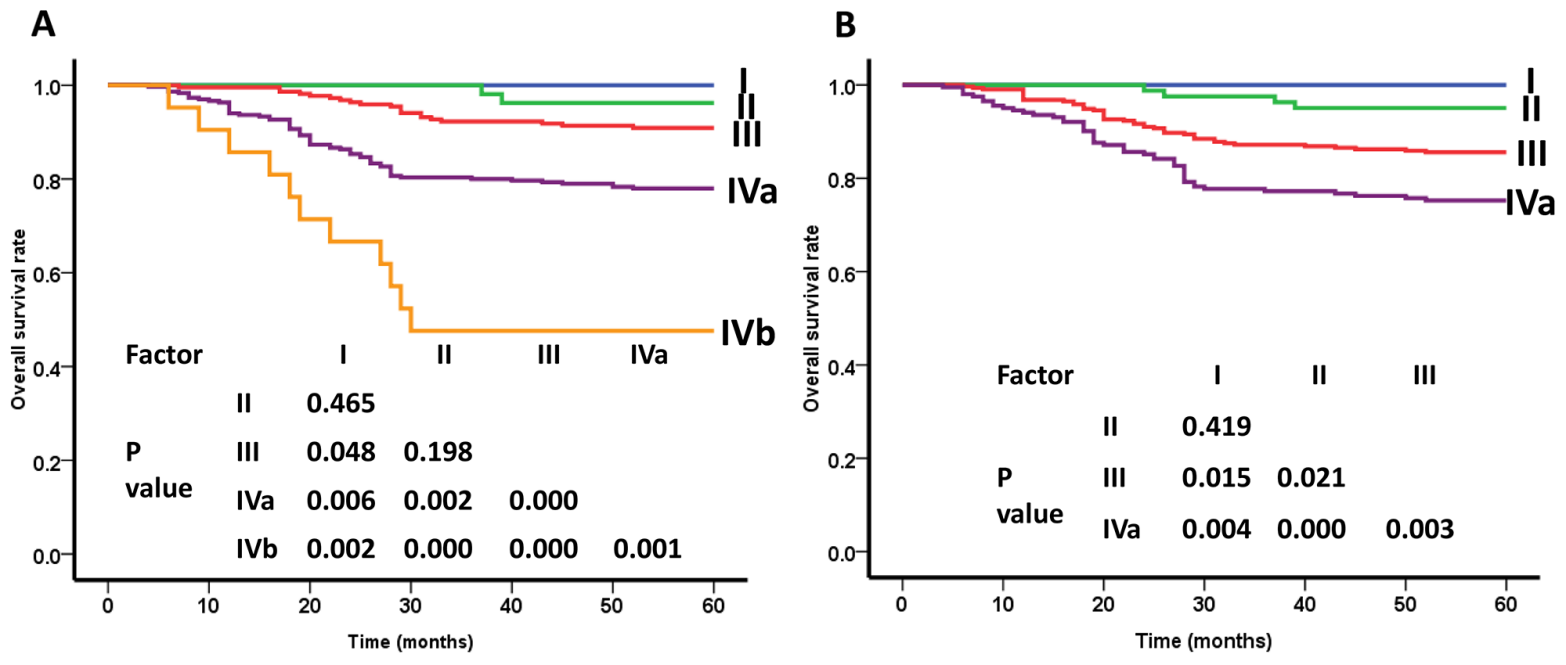
Overall survival (OS) curves of nasopharyngeal carcinoma patients for different clincal stage groups **(A)** As defined by the 7th edition UICC/AJCC staging system, the OS curves showed no significant difference between stages I and II, and between stages II and III (P=0.465 and P=0.198). **(B)** As defined by the 8th edition UICC/AJCC staging system, the OS curves showed good segregation between stages II and III, and between stages III and IVa (P<0.05), but not between stages I and II (P=0.419).

Using the 8^th^ edition of the UICC/AJCC staging system, the overall survival (OS) rates of patients with stages I, II, III, and IVa were 100%, 95.1%, 85.6%, and 75.2%, respectively. The survival curves showed good segregation between stages II and III, and between stages III and IVa, but not between stages I and II (P=0.419). When the 7^th^ edition was used, the OS curves showed no significant difference between stages I and II, and between stages II and III (P=0.465 and P=0.198). Thus, the total difference between stages I and IV was slightly larger when the 8^th^ edition, rather than the 7^th^ edition was used (Figure 3).

### Risk difference

To identify the risk difference between adjacent stages, the hazard ratios of the survival analysis between the 7^th^ and 8^th^ editions of the UICC/AJCC staging systems were used (Table 2). For the T classifications, the included parameters were as follows: histology (WHO I+III vs. WHO II), chemotherapy (yes vs. no), gender (male vs. female), age (>50 yrs vs. <=50 yrs), and N classifications. The LRFS was evaluated as an endpoint. The hazard ratios (HRs) between T1 and T2, T2 and T3, and T1 and T3, were not significantly different between the two classification systems. When the T classifications were regrouped as two stages (T1+T2+T3 and T4), the difference between adjacent T stages was slightly larger when the 7^th^ edition system was used compared to the 8^th^ edition. In the N classifications, the following covariables were included in the cox proportion hazards model by backward elimination of non-significant explanatory variables: histology (WHO I+III vs. WHO II), age (>50 yrs vs. <=50 yrs), gender (male vs. female), chemotherapy (yes vs. no), and T classifications; the DMFS was chosen as an endpoint. Both systems showed that a higher N stage was associated with a poor DMFS rate, but this was not observed between N3a and N3b (P=0.907), as defined by the 7^th^ edition staging system. In regards to stage grouping, the following covariables were included in the cox proportion hazards model: histology (WHO I+III vs. WHO II), age (>50 yrs vs. <=50 yrs), gender (male vs. female), chemotherapy (yes vs. no), T classifications, and N classifications; the OS was evaluated as an endpoint, The risk differentials between each adjacent stage of the 8^th^ edition of the UICC/AJCC staging system were all higher than in the 7^th^ edition. Moreover, when the 8^th^ edition system was used, we found significant risk differentials when stage III was compared with stages II and IVa. Although the HRs between stages I and II did not reach statistical significance, they showed a relatively better segregated pattern for each adjacent stage in the 8^th^ edition. In contrast, in the 7^th^ UICC/AJCC staging system, these HRs exhibited no statistical significance in OS between I and II, and between II and III (HR = 0.554, 95% CI = 0.049-6.022, P=0.619; HR = 2.509, 95% CI = 0.587-10.736, P=0.215).

**Table 2:** Risk differentiation compared by Cox regression analyses between the two staging systems

		The seventh edition UICC/AJCC system		The eighth edition UICC/AJCC system	
		HR (95% CI)	P value	HR (95% CI)	P value
**5-yr LRFS**	T1 VS T2	0.257(0.016-4.116)	0.337	0.630(0.070-5.639)	0.680
	T2 VS T3	2.840(0.258-31.319)	0.394	2.239(0.749-6.698)	0.149
	T3 VS T4	2.583(1.583-4.214)	0.000	2.432(1.364-4.336)	0.003
	T1+T2+T3 VS T4	5.085(1.846-14.008)	0.002	3.070(1.813-5.197)	0.001
**5-yr DMFS**	N0 VS N1	2.933(1.470-5.849)	0.002	2.933(1.470-5.849)	0.002
	N1 VS N2	2.040(1.291-3.224)	0.002	2.040(1.291-3.224)	0.002
	N2 VS N3	9.473(1.290-9.557)	0.027	9.677(1.300-72.047)	0.027
	N3a VS N3b	0.911(0.193-4.303)	0.907		
**5-yr OS**	I VS II	0.544(0.049-6.022)	0.619	0.662(0.074-5.920)	0.712
	II VS III	2.509(0.587-10.736)	0.215	3.110(1.118-8.647)	0.030
	III VS IVa	1.799(1.200-2.697)	0.004	2.628(1.592-4.338)	0.000
	IVa VS IVb	2.905(1.532-5.508)	0.001		

## DISCUSSION

An ideal TNM staging system should satisfy the following conditions [13]: (1) The survival rates within each stage should be similar; (2) The survival rates should differ significantly between adjacent stages; (3) A relatively balanced distribution proportion should exist between each stage; (4) Accurate evaluation of the prognosis of malignant tumors should occur. Recently, UICC and AJCC committees have advocated the 8^th^ UICC/AJCC edition for nasopharyngeal carcinoma (NPC) staging; this edition has been generated in response to recent clinical data and improved management approaches.

This study is the first to use the 8^th^ edition UICC/AJCC TNM staging system for NPC. Our results showed that the 5-year OS, DFS, LRFS, and DMFS rates of the NPC patients were 81.5%, 80.1%, 86.0%, and 81.1%, respectively. Chen et al. [14] reported 512 NPC patients who were treated with IMRT and 334 patients who received chemotherapy; the 4-year OS, DMFS, DFS, and LRFS rates were 85.9%, 85.7%, 79.4%, and 94.1%, respectively. This is consistent with our results. With improvements that have resulted from modern therapeutic technologies, the prognosis of NPC patients has dramatically improved.

According to the 7^th^ edition staging system for NPC, a variety of studies have reported that the survival rates between each T stage in NPC patients are not significantly different after treatment with IMRT [15–17]. Zhao et al. [18] retrospectively analyzed 527 patients who were treated with IMRT and found that the 5-year LFSR rates between stage T1 and T2 (X^2^=3.540, P=0.060), T2 and T3 (X^2^=0.684, P=0.408), and T3 and T4 (X^2^=3.264, P=0.071) were not significantly different. Jiang F et al. [19] analyzed 720 NPC patients who were treated with definitive IMRT and found that the LRFS rates among T1 to T3 were not significantly different. Tham et al. [20] also reported that the LRFS rates among T1, T2, and T3 patients were not significantly different after excellent local-regional control of NPC treated with IMRT. Zhou Q et al. [21] conducted a retrospective analysis of 358 patients with stage T3/T4 NPC who had received IMRT and found that the T category was neither a significant factor for OS/LRFS nor an independent prognostic factor for OS/LRFS/DMFS/DFS. Based on the above results, Pan et al. [22] proposed the 8^th^ edition of the UICC/AJCC staging system for NPC, and analyzed 1609 patients who were treated with IMRT at two clinical centers. They found that tumors invading medial pterygoid and/or lateral pterygoid muscle had similar OS to tumors with extension to parapharyngeal space and prevertebral muscle (P=0.44). Therefore, they proposed a down-staging of the medial pterygoid and lateral pterygoid muscle to T2 in the 8^th^ edition of the UICC/AJCC staging system. However, after they staged the patients according to the 8^th^ edition of the UICC/AJCC staging system, they found that the OS and DMFS curves between T1 and T2 (P=0.19, P=0.16), or the LRFS and DMFS curves between T2 and T3 (P=0.60, P=0.11) did not significantly differ. Our research also found that the difference in LRFS rates among T1, T2, and T3 patients was not significant according to the 8^th^ edition of the UICC/AJCC staging system, indicating that the hazard discrimination among T1-3 patients was diminished. Moreover, when we regrouped the T classifications as two groups (T1+T2+T3 vs. T4), the HRs of 8^th^ editions (HR = 3.070, 95%CI = 1.813-5.197, P=0.001) between adjacent T stages were smaller than in the 7th editions (HR = 5.085, 95% CI = 1.846-14.008, P=0.002). Consequently, our previous study had suggested that the T classifications should be subdivided into two stages rather than four stages [11, 12]. Since our current study shows that there is no statistical difference between adjacent T stages using the 8^th^ edition of the UICC/AJCC staging system, the T classification using the 8^th^ edition remains controversial.

Unlike the 7^th^ edition of the UICC/AJCC staging system, the 8^th^ edition has merged N3a and N3b stages. This seems more reasonable, since several studies have found that the DMFS rates between N3a and N3b stages are not significantly different [22–24]. In a retrospective analysis of 985 newly diagnosed NPC patients, Lee et al. [25] found that in patients who did not have distant metastases, but received IMRT, the difference in the 5-year regional FFR among N0-N2 patients and between N3a and N3b patients was insignificant. Our previous study [11] has also indicated that according to the 7^th^ edition staging system, there is no difference in DMFS between N3a and N3b stages. Our current results show that using the 8^th^ edition of the UICC/AJCC staging system, the DMFS curves between N0 and N1, N1 and N2, and N2 and N3 exhibit good separation, while the HRs for DMFS among adjacent N stages significantly differ, indicating that N staging of the 8^th^ edition is practical and can accurately predict the NPC patients prognosis.

Using the 8^th^ edition, the OS rates of patients with stages I, II, III, and IVa disease exhibit a better distribution compared with the 7^th^ edition. After stage IVb was down-staged to IVa, the OS curves between stages III and IVa are more separated. Additionally, the HRs for OS between stages II and III, and between III and IVa differ significantly. However, the differences of OS between stages I and II did not reach statistical significance (X^2^=0.654, P=0.419). Lee et al. [25] analyzed 985 patients staged by the 7^th^ edition system and found that the differences in HRs between stages I and II, and between stages IVa and IVb were minimal, which suggested that stage II of the 7^th^ edition should be down-staged to stage I and that the stages IVa and IVb should be merged. Pan [26] argued that although the LRFS rates between stages I and II were similar and the number of patients with T1N0 was low, it was not rational to combine stages I and II into stage I, because stage II patients might benefit from chemotherapy. Therefore, the down-staging of IVb to IVa in the 8^th^ edition was appropriate. Su et al. [27] have demonstrated that while concurrent chemoradiotherapy (CRT) does not improve survival of patients with stage II NPC, it increases the occurrence of acute toxicity reactions. A meta-analysis by Ma et al. [28] has also shown that compared to IMRT alone, CRT does not improve OS, LRRFS, or DMFS for the stage II patients, and is associated with a higher frequency of grade 3-4 leukopenia and thrombocytopenia than IMRT alone. Thus, they concluded that IMRT alone was better than CRT for patients with stage II NPC. Similarly, in our study, 34.8% (30/46) of stage II defined by the 7^th^ edition, and 67.0% (61/91) of stage II defined by the 8^th^ edition, received platinum-based chemotherapy. Some stage II patients probably benefit from chemotherapy, resulting in the similar 5-year OS curves for stages I and II. However, the multivariate analysis by cox proportion hazards model also showed that the HRs between stages I and II did not reach statistical significance after chemotherapy. Therefore, more studies are needed to determine whether the current stages I and II should be merged.

With changes to the 8^th^ edition, 7.7% (47/608) of patients were down-staged from T4 to T2, 10.2% (62/608) were down-staged from T4 to T3, 7.4% (45/608) were down-staged from stages IVa to II, and 10.6% (64/608) were down-staged from stages IVa to III. Compared with previous staging systems, the proportion of cases that was distributed among adjacent stages as classified by the 8^th^ edition seemed more balanced.

However, there are some limitations of this study. First, the sample size was relatively small for evaluation of NPC prognoses. Therefore, further studies including patients from multiple centers are warranted. Second, the current study of the UICC/AJCC staging system for NPC mostly relied on the anatomic extension of the primary tumor diagnosed by imaging methods, pathologic and histologic features, and clinical symptoms. However, future UICC/AJCC staging systems should consider also genetic, biologic, and molecular factors relevant for the pathogenesis of NPC.

In conclusion, our data indicate that the 8^th^ edition presents a better stage distribution and a more accurate segregation of survival rates than the 7^th^ edition. However, the 8^th^ edition system still has some limitations, i.e., the survival risk between adjacent T1-T3 stages does not significantly differ, and stages I and II lack statistical difference. Thus, with improving NPC outcomes, further simplification of the staging system is warranted.

## MATERIALS AND METHODS

### Patient characteristics

Between January 2008 and March 2010, 608 IMRT-treated patients with biopsy-proven, non-metastatic NPC were selected from the first affiliated hospital of Guangxi Medical University, China. Among them, 427 were males, and 181 were females. The median age was 46 years (range, 19-74 years of age). Before treatment, all patients were required to undergo a detailed physical examination, routine blood examination, nasopharyngeal fiberscope examination, chest X-ray or CT, abdominal ultrasound, and MRI of the nasopharynx and neck. For patients with N2-3 disease, additional bone scanning was performed.

### Clinical staging

The MRI images of each patient were independently reviewed on the PACS system by two physicians, and each patient was staged according to the criteria of the 7^th^ and the 8^th^ edition of the UICC/AJCC staging system. If a consensus could not be reached, the research team then defined the stage according to the staging system and other information, such as cranial nerve palsy and size of the lymph nodes. Written informed consent was obtained from all patients, and the study was approved by the Institutional Review Board (IRB) of Guangxi Medical University. Using the 8^th^ edition of the UICC/AJCC Staging System, 1.3% (8/608), 15.0% (91/608), 46.7% (296/608), and 35.0% (213/608) were classified as stages I, II, III, and IVa, respectively.

### Treatment

#### IMRT

The target delineation was in accordance with the International Commission on Radiation Units and Measurements Reports 50 and 62. The gross tumor volume (GTV) included the primary tumor site (GTVnx) and the metastatic cervical lymph node (GTVnd). The clinical target volume (CTV) was adjusted according to the presence of tumor invasion. The prescription doses were as follows: PGTVnx (68-74 Gy), PTVnd (66-70 Gy), PTV1 (60-66 Gy), and PTV2 (50-56 Gy). All targets were treated once daily and 5 times weekly, for a total fraction of 30-33 Gy. The details of the IMRT treatment were reported in our previous studies [11, 12].

#### Chemotherapy

A total of 93.0% (570/608) of patients received platinum-based chemotherapy. Of these, 44.9% (273/608) received concurrent chemotherapy, 37.0% (225/608) received induction plus concurrent chemotherapy, 7.57% (46/608) received concurrent plus adjuvant chemotherapy, 4.11% (25/608) received induction, concurrent, and adjuvant chemotherapy, and 0.16% (1/608) received induction chemotherapy.

### Statistical analysis

The follow-up time was calculated from the date of treatment completion to the date of last contact or death. Time to failure was calculated from the date of treatment completion to the date of the relevant event. The Statistical Package for the Social Sciences, version 18.0 software (SPSS, Chicago, IL, USA) was used for all statistical analyses. A Kappa analysis, the Kaplan-Meier method, and the Log-rank test were applied to compare the distribution consistency in patients with different disease stages, to estimate the survival rates, and to test the differences in survival rates, respectively. The endpoints included overall survival (OS), disease-free survival (DFS), local relapse-free survival (LRFS), and distant metastasis-free survival (DMFS). A two-tailed p value of <0.05 was considered significant.

## Abbreviations

NPC: Nasopharyngeal carcinoma
IMRT: intensity-modulated radiotherapy
OS: overall survival
DFS: disease-free survival
LRFS: local relapse-free survival
DMFS: distant metastasis-free survival
